# Abrupt‐Onset Dementia: Differential Diagnosis and Clinical Characteristics of a Rare Condition

**DOI:** 10.1002/alz.087411

**Published:** 2025-01-03

**Authors:** Yoav D Piura, Tara Brigham, Moain Abu Dabrh, Gregory S Day

**Affiliations:** ^1^ Mayo Clinic, Jacksonville, FL USA

## Abstract

**Background:**

The term rapidly progressive dementia (RPD) may be applied to patients with precipitous declines in cognitive function resulting in dementia within one year or complete incapacitation within two‐years of symptom onset. Although most patients present with subacute, progressive declines, selected patients develop complete incapacitation within seven days of symptom onset. The differential diagnosis and clinical characteristics of patients with abrupt‐onset dementia are not known.

**Method:**

187 patients with RPD were prospectively enrolled from February 2016 to September 2023 in a study of RPD at Mayo Clinic (Jacksonville, Florida) and Washington University (Saint Louis, Missouri). Patients with ≤7 days from first symptom to onset of RPD were labelled as abrupt onset. Patient characteristics, results of investigations, and causes of abrupt‐onset dementia were further characterized. We further explored the causes of abrupt‐onset dementia in the extant literature to refine the differential diagnosis and approach to evaluation and management of these patients.

**Result:**

Two patients in our study of RPD had abrupt‐onset RPD (2/187, 1.1%), attributed to autoimmune and vascular. An additional 63 cases were identified through comprehensive meta‐narrative review of the literature (40 publications). Causes of abrupt‐onset dementia include vascular, infectious, inflammatory, and toxic. Compromise of structures within the Papez circuit was a common unifying feature common across patients with varying etiologies, informing the localization of abrupt‐onset dementia. 20% cases had potentially treatment‐responsive causes of abrupt‐onset dementia.

**Conclusion:**

Cases of abrupt‐onset dementia are rare overall and associated with a limited differential diagnosis. Evaluation of patients with abrupt‐onset dementia should prioritize testing for vascular, autoimmune, infectious, and toxic causes. Early‐recognition of patients with potentially treatment‐responsive causes of dementia may promote earlier intervention leading to better outcomes in selected patients.

